# 2-Chloro-5-(chloro­meth­yl)pyridine

**DOI:** 10.1107/S1600536811000821

**Published:** 2011-01-12

**Authors:** Zhi-Qiang Feng, Xiao-Li Yang, Yuan-Feng Ye, Huai-Qing Wang, Ling-Yun Hao

**Affiliations:** a School of Material Engineering, Jinling Institute of Technology, Nanjing 211169, People’s Republic of China

## Abstract

The title compound, C_6_H_5_Cl_2_N, is almost planar, with an r.m.s. deviation of 0.0146 Å for all atoms except for the 5-choloromethyl Cl atom. The offset Cl atom lies above this plane with a Cl—C—C angle of 111.11 (17)°. In the crystal, mol­ecules are connected *via* inter­molecular C—H⋯N hydrogen bonds, forming dimers.

## Related literature

For the synthetic procedure, see: Nishihara *et al.* (1993 ▶). For bond-length data, see: Allen *et al.* (1987 ▶). The title compound is an inter­mediate in the synthesis of imidacloprid [systematic name (*E*)-1-(6-chloro-3-pyridyl­meth­yl)-*N*-nitro­imidazolidin-2-yl­idene­amine], see: Shroff *et al.* (2007 ▶).
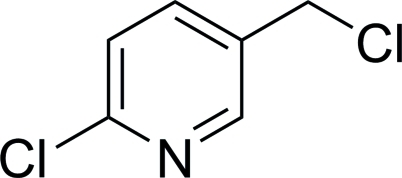


## Experimental

### 

#### Crystal data

C_6_H_5_Cl_2_N
*M* *_r_* = 162.01
Monoclinic, 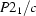

*a* = 4.0770 (8) Å
*b* = 10.322 (2) Å
*c* = 16.891 (3) Å
β = 95.95 (3)°
*V* = 707.0 (2) Å^3^
*Z* = 4
Mo *K*α radiation
μ = 0.82 mm^−1^
*T* = 293 K
0.30 × 0.20 × 0.20 mm

#### Data collection

Enraf–Nonius CAD-4 diffractometer
Absorption correction: ψ scan (North *et al.*, 1968 ▶) *T*_min_ = 0.791, *T*_max_ = 0.853
2886 measured reflections
1299 independent reflections
1028 reflections with *I* > 2σ(*I*)
*R*_int_ = 0.049
3 standard reflections every 200 reflections  intensity decay: 1%

#### Refinement

*R*[*F* ^2^ > 2σ(*F* ^2^)] = 0.037
*wR*(*F* ^2^) = 0.129
*S* = 1.00
1299 reflections
83 parameters
H-atom parameters constrained
Δρ_max_ = 0.19 e Å^−3^
Δρ_min_ = −0.18 e Å^−3^

### 

Data collection: *CAD-4 Software* (Enraf–Nonius, 1985 ▶); cell refinement: *CAD-4 Software*; data reduction: *XCAD4* (Harms & Wocadlo, 1995 ▶); program(s) used to solve structure: *SHELXS97* (Sheldrick, 2008 ▶); program(s) used to refine structure: *SHELXL97* (Sheldrick, 2008 ▶); molecular graphics: *SHELXTL* (Sheldrick, 2008 ▶); software used to prepare material for publication: *SHELXTL*.

## Supplementary Material

Crystal structure: contains datablocks I, global. DOI: 10.1107/S1600536811000821/bq2267sup1.cif

Structure factors: contains datablocks I. DOI: 10.1107/S1600536811000821/bq2267Isup2.hkl

Additional supplementary materials:  crystallographic information; 3D view; checkCIF report

## Figures and Tables

**Table 1:** Hydrogen-bond geometry (Å, °)

*D*—H⋯*A*	*D*—H	H⋯*A*	*D*⋯*A*	*D*—H⋯*A*
C6—H6*A*⋯N^i^	0.97	2.57	3.453 (3)	151

Symmetry code: (i) 
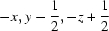
.

**Fig. 1.**
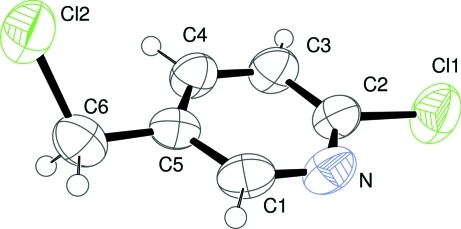
The molecular structure of (I), with the atom-numbering scheme. Displacement ellipsoids are drawn at the 50% probability level.

**Fig. 2.**
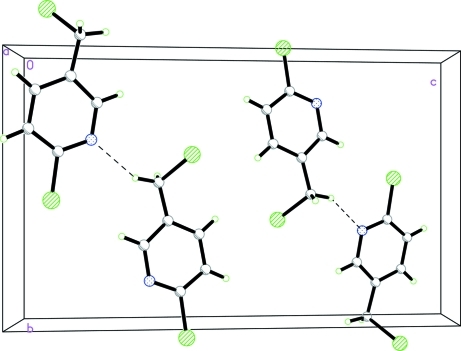
A packing diagram for (I). C—H···N hydrogen bonds are shown by dashed lines.

## supplementary crystallographic information

### Comment

The tittle compound, 2-chloro-5-(chloromethyl)pyridine (I), is an important intermediate for the synthesis of imidacloprid (Shroff *et al.*, 2007) and we report here its crystal structure.

The molecular structure of (I) is shown in Fig. 1. The bond lengths and angles are within normal ranges (Allen *et al.*, 1987). In the crystal structure, the molecules were connected together *via* weak C—H···N intermolecular hydrogen bonds forming dimers, which seems to be effective in the stabilization of the crystal structure.

### Experimental

The title compound, (I) was prepared by a method reported in literature (Nishihara *et al.*, 1993). The crystals were obtained by dissolving (I) (0.2 g, 1.2 mmol) in ethanol (25 ml) and evaporating the solvent slowly at room temperature for about 5 d.

### Refinement

H atoms were positioned geometrically (C–H = 0.93–0.97 Å) and refined using a riding model with *U*_iso_(H) = *xU*_eq_(C, O), where *x* = 1.5 for methyl and oxygen H-atoms and *x* = 1.2 for all other H-atoms.

### Crystal data

**Table:**

C_6_H_5_Cl_2_N	*F*(000) = 328
*M**_r_* = 162.01	*D*_x_ = 1.522 Mg m^−^^3^
Monoclinic, *P*2_1_/*c*	Mo *K*α radiation, λ = 0.71073 Å
Hall symbol: -P 2ybc	Cell parameters from 25 reflections
*a* = 4.0770 (8) Å	θ = 10–14°
*b* = 10.322 (2) Å	µ = 0.82 mm^−^^1^
*c* = 16.891 (3) Å	*T* = 293 K
β = 95.95 (3)°	Block, colorless
*V* = 707.0 (2) Å^3^	0.30 × 0.20 × 0.20 mm
*Z* = 4	

### Data collection

**Table:**

Enraf–Nonius CAD-4 diffractometer	1028 reflections with *I* > 2σ(*I*)
Radiation source: fine-focus sealed tube	*R*_int_ = 0.049
graphite	θ_max_ = 25.4°, θ_min_ = 2.3°
ω/2θ scans	*h* = 0→4
Absorption correction: ψ scan (North *et al.*, 1968)	*k* = −12→12
*T*_min_ = 0.791, *T*_max_ = 0.853	*l* = −20→20
2886 measured reflections	3 standard reflections every 200 reflections
1299 independent reflections	intensity decay: 1%

### Refinement

**Table:**

Refinement on *F*^2^	Secondary atom site location: difference Fourier map
Least-squares matrix: full	Hydrogen site location: inferred from neighbouring sites
*R*[*F*^2^ > 2σ(*F*^2^)] = 0.037	H-atom parameters constrained
*wR*(*F*^2^) = 0.129	*w* = 1/[σ^2^(*F*_o_^2^) + (0.090*P*)^2^] where *P* = (*F*_o_^2^ + 2*F*_c_^2^)/3
*S* = 1.00	(Δ/σ)_max_ < 0.001
1299 reflections	Δρ_max_ = 0.19 e Å^−^^3^
83 parameters	Δρ_min_ = −0.18 e Å^−^^3^
0 restraints	Extinction correction: *SHELXL97* (Sheldrick, 2008), Fc^*^=kFc[1+0.001xFc^2^λ^3^/sin(2θ)]^-1/4^
Primary atom site location: structure-invariant direct methods	Extinction coefficient: 0.025 (5)

### Special details

**Table:**

Geometry. All e.s.d.'s (except the e.s.d. in the dihedral angle between two l.s. planes) are estimated using the full covariance matrix. The cell e.s.d.'s are taken into account individually in the estimation of e.s.d.'s in distances, angles and torsion angles; correlations between e.s.d.'s in cell parameters are only used when they are defined by crystal symmetry. An approximate (isotropic) treatment of cell e.s.d.'s is used for estimating e.s.d.'s involving l.s. planes.
Refinement. Refinement of *F*^2^ against ALL reflections. The weighted *R*-factor *wR* and goodness of fit *S* are based on *F*^2^, conventional *R*-factors *R* are based on *F*, with *F* set to zero for negative *F*^2^. The threshold expression of *F*^2^ > σ(*F*^2^) is used only for calculating *R*-factors(gt) *etc*. and is not relevant to the choice of reflections for refinement. *R*-factors based on *F*^2^ are statistically about twice as large as those based on *F*, and *R*- factors based on ALL data will be even larger.

### Fractional atomic coordinates and isotropic or equivalent isotropic displacement parameters (Å^2^)

**Table:**

	*x*	*y*	*z*	*U*_iso_*/*U*_eq_	
N	0.1376 (5)	0.3311 (2)	0.19100 (11)	0.0614 (5)	
Cl1	0.2101 (2)	0.54073 (7)	0.11039 (4)	0.0867 (4)	
C1	0.1642 (6)	0.2038 (2)	0.20387 (12)	0.0573 (6)	
H1A	0.0900	0.1710	0.2501	0.069*	
Cl2	0.11280 (19)	−0.12135 (6)	0.09764 (4)	0.0757 (3)	
C2	0.2433 (6)	0.3739 (2)	0.12537 (14)	0.0559 (6)	
C3	0.3774 (7)	0.2985 (2)	0.06990 (14)	0.0598 (6)	
H3A	0.4474	0.3344	0.0240	0.072*	
C4	0.4038 (6)	0.1686 (2)	0.08483 (13)	0.0558 (6)	
H4A	0.4954	0.1144	0.0491	0.067*	
C5	0.2944 (5)	0.1178 (2)	0.15315 (12)	0.0490 (5)	
C6	0.3244 (6)	−0.0224 (2)	0.17406 (15)	0.0621 (6)	
H6A	0.2322	−0.0375	0.2239	0.075*	
H6B	0.5556	−0.0464	0.1812	0.075*	

### Atomic displacement parameters (Å^2^)

**Table:**

	*U*^11^	*U*^22^	*U*^33^	*U*^12^	*U*^13^	*U*^23^
N	0.0734 (14)	0.0648 (12)	0.0481 (10)	−0.0050 (10)	0.0169 (9)	−0.0118 (9)
Cl1	0.1250 (8)	0.0567 (4)	0.0825 (6)	0.0007 (4)	0.0292 (5)	−0.0030 (3)
C1	0.0626 (14)	0.0737 (14)	0.0374 (11)	−0.0081 (12)	0.0137 (10)	−0.0006 (9)
Cl2	0.0946 (6)	0.0576 (4)	0.0772 (5)	−0.0046 (3)	0.0203 (4)	−0.0059 (3)
C2	0.0645 (14)	0.0562 (13)	0.0477 (11)	−0.0062 (10)	0.0089 (11)	−0.0047 (9)
C3	0.0761 (16)	0.0632 (13)	0.0426 (11)	−0.0057 (12)	0.0187 (11)	0.0020 (10)
C4	0.0640 (14)	0.0614 (13)	0.0444 (12)	0.0020 (11)	0.0166 (10)	−0.0048 (9)
C5	0.0445 (11)	0.0614 (12)	0.0410 (10)	−0.0022 (10)	0.0044 (9)	0.0011 (9)
C6	0.0638 (15)	0.0686 (14)	0.0545 (13)	0.0052 (12)	0.0088 (11)	0.0120 (11)

### Geometric parameters (Å, °)

**Table:**

N—C2	1.307 (3)	C3—C4	1.367 (3)
N—C1	1.334 (3)	C3—H3A	0.9300
Cl1—C2	1.743 (3)	C4—C5	1.383 (3)
C1—C5	1.378 (3)	C4—H4A	0.9300
C1—H1A	0.9300	C5—C6	1.491 (3)
Cl2—C6	1.795 (3)	C6—H6A	0.9700
C2—C3	1.375 (3)	C6—H6B	0.9700
			
C2—N—C1	116.3 (2)	C3—C4—H4A	120.0
N—C1—C5	124.3 (2)	C5—C4—H4A	120.0
N—C1—H1A	117.9	C1—C5—C4	116.9 (2)
C5—C1—H1A	117.9	C1—C5—C6	120.3 (2)
N—C2—C3	125.1 (2)	C4—C5—C6	122.7 (2)
N—C2—Cl1	115.46 (18)	C5—C6—Cl2	111.11 (17)
C3—C2—Cl1	119.40 (18)	C5—C6—H6A	109.4
C4—C3—C2	117.3 (2)	Cl2—C6—H6A	109.4
C4—C3—H3A	121.3	C5—C6—H6B	109.4
C2—C3—H3A	121.3	Cl2—C6—H6B	109.4
C3—C4—C5	120.0 (2)	H6A—C6—H6B	108.0
			
C2—N—C1—C5	0.2 (4)	N—C1—C5—C4	0.1 (4)
C1—N—C2—C3	−0.1 (4)	N—C1—C5—C6	177.9 (2)
C1—N—C2—Cl1	−179.33 (18)	C3—C4—C5—C1	−0.6 (3)
N—C2—C3—C4	−0.4 (4)	C3—C4—C5—C6	−178.3 (2)
Cl1—C2—C3—C4	178.9 (2)	C1—C5—C6—Cl2	123.5 (2)
C2—C3—C4—C5	0.7 (4)	C4—C5—C6—Cl2	−58.9 (3)

### Hydrogen-bond geometry (Å, °)

**Table:**

*D*—H···*A*	*D*—H	H···*A*	*D*···*A*	*D*—H···*A*
C6—H6A···N^i^	0.97	2.57	3.453 (3)	151

Symmetry codes: (i) −*x*, *y*−1/2, −*z*+1/2.
